# Contrasting effects of preexisting hyperglycemia and higher body size on hospital mortality in critically ill patients: a prospective cohort study

**DOI:** 10.1186/1472-6823-14-50

**Published:** 2014-06-17

**Authors:** Marina Verçoza Viana, Rafael Barberena Moraes, Amanda Rodrigues Fabbrin, Manoella Freitas Santos, Vanessa Bielefeldt Leotti Torman, Silvia Regina Vieira, Jorge Luiz Gross, Luis Henrique Canani, Fernando Gerchman

**Affiliations:** 1 Intensive Care Unit, Division of Endocrinology, Hospital de Clínicas de Porto Alegre, Federal University of Rio Grande do Sul, Porto Alegre, Brazil; 2 Division of Endocrinology, Hospital de Clínicas de Porto Alegre, Federal University of Rio Grande do Sul, Porto Alegre, Brazil; 3 Department of Statistics, Federal University of Rio Grande do Sul, and Research and Post-Graduation Group, Hospital de Clínicas de Porto Alegre, Porto Alegre, Brazil

**Keywords:** Diabetes mellitus, Glycated hemoglobin, Obesity, Intensive care unit, Mortality

## Abstract

**Background:**

Obesity and diabetes mellitus are well-defined risk factors for cardiovascular mortality. The impact of antecedent hyperglycemia and body size on mortality in critical ill patients in intensive care units (ICUs) may vary across their range of values. Therefore, we prospectively analyzed the relationship between in-hospital mortality and preexisting hyperglycemia and body size in critically ill ICU patients to understand how mortality varied among normal, overweight, and obese patients and those with low, intermediate, and high glycated hemoglobin (HbA_1c_) levels.

**Methods:**

Medical history, weight, height, physiologic variables, and HbA_1c_ were obtained during the first 24 h for patients who were consecutively admitted to the high complexity ICU of Hospital de Clínicas de Porto Alegre, Brazil, from April to August 2011. The relationships between mortality and obesity and antecedent hyperglycemia were prospectively analyzed by cubic spline analysis and a Cox proportional hazards model.

**Results:**

The study comprised 199 patients. The overall hospital mortality rate was 43.2% during a median 16 (8–28) days of follow-up. There was a progressive risk of in-hospital mortality with higher HbA_1c_ levels, with the relationship becoming significant at HbA_1c_ >9.3% compared with lower levels (hazard ratio 1.74; 95% confidence interval with Bonferroni correction 1.49–2.80). In contrast, mean body mass index (BMI) was higher in survivors than in nonsurvivors (27.2 kg/m^2^ ± 7.3 vs. 24.7 kg/m^2^ ± 5.0 *P* = 0.031, respectively). Cubic spline analysis showed that these relationships differed nonlinearly through the spectrum of BMI values. In a Cox proportional hazards model adjusted for Acute Physiology and Chronic Health Evaluation II score and HbA_1c_, the risk of in-hospital mortality progressively decreased with increasing BMI (BMI <20 vs. 20–23.9 kg/m^2^, *P* = 0.032; BMI <20 vs. 24–34.9 kg/m^2^, *P* = 0.010; BMI <20 vs. ≥35 kg/m^2^, *P* = 0.032).

**Conclusions:**

Our findings suggest that significant hyperglycemia prior to ICU admission is a risk factor for in-hospital mortality. Conversely, increasing BMI may confer an advantageous effect against mortality in critical illness independently of previous glycemic control.

## Background

Diabetes mellitus (DM) and obesity are well-defined risk factors for cardiovascular disease and mortality [1,2]. Both diseases have assumed epidemic proportions in the last few decades [1,3]. As a consequence, the increasing rates of admission of patients with obesity and/or DM to intensive care units (ICUs) have become a concern for intensivists [4].

A large body of evidence has defined poor glycemic control as a negative prognostic factor in critical illness [5,6]. This association is especially true in patients without a past known history of DM, but is poorly defined in those with known DM [7-9]. It is also unclear how glycemic control before ICU admission affects prognosis in critically ill patients.

The measurement of glycated hemoglobin (HbA_1c_) levels is widely employed to estimate average glucose levels over a 2- to 3-month period, providing a method for the assessment of past glycemic control [10]. Recently, the American Diabetes Association (ADA) proposed the use of HbA_1c_ levels for the diagnosis of prediabetes and DM [11]. Because HbA_1c_ reflects past glycemic control, it can be used to understand the relationship between prior hyperglycemia and morbidity and mortality in recently admitted critically ill patients [10,11]. Recent data have linked higher HbA_1c_ levels to increased ICU mortality [12].

Obesity is considered a risk factor for the development of DM [1,3]. The impact of obesity as a prognostic factor in critically ill patients is controversial, and possibly influenced by the presence of DM [13]. Findings to date are not conclusive, with some studies reporting no association between obesity and mortality [14,15], and others finding that low body mass index (BMI) is associated with increased mortality [15-17], or that excess weight is a negative prognostic factor [18,19]. Two meta-analyses pooling data on studies evaluating the impact of obesity on outcomes in ICU patients did not find an association between obesity and mortality in critical illness [20,21].

However, data analyzing the effect of glycemic status and body size on survival in ICU patients across the spectrum of body size and glycemic status may give the opportunity to better understand the complex relationship between hyperglycemia, excess weight, and survival in critical illness. To address this issue, we investigated whether preexisting hyperglycemia and body size are determinants of morbidity and mortality in patients recently admitted to the ICU.

## Methods

### Study design

This was a prospective observational study carried out in the ICU of Hospital de Clínicas de Porto Alegre, an 850-bed teaching hospital of Federal University of Rio Grande do Sul, Brazil. Written informed consent was obtained from each participant (or surrogate), and the study was approved by Research Ethics Committee of Hospital de Clínicas de Porto Alegre. No patients (or surrogate) refused to participate in the study.

### Study population and data source

Consecutive medical and surgical patients aged ≥18 years who were first admitted to the ICU between April 1, 2011, and August 22, 2011, were eligible for inclusion. Patients with hyperglycemic crisis (diabetic ketoacidosis or hyperosmolar hyperglycemic states) and those with hemoglobinopathies were excluded.

Clinical and demographic data were collected at baseline, including a detailed past medical history, data regarding corticosteroid and vasopressor therapy, mechanical ventilation support, and renal replacement therapy. Physiologic and laboratory data were collected for calculation of Acute Physiology and Chronic Health Evaluation (APACHE) II and Sequential Organ Failure Assessment (SOFA) scores [22,23]. Supine height (cm) and weight (kg), as measured routinely on ICU admission, were used to calculate BMI, expressed as weight (kg)/height^2^ (m).

Glucose (hexokinase assay), ultrasensitive C-reactive protein (enzymatic colorimetric assay), lactate (turbidimetric method), and HbA_1c_ (high-performance liquid chromatography using Tosoh 2.2 Plus HbA_1c_; Tosoh Corporation, Tokyo, Japan) were measured within 24 h of ICU admission. The HbA_1c_ method was certified by the National Glycohemoglobin Standardization Program (NGSP) (http://www.ngsp.org/critsumm.asp) and values were International Federation of Clinical Chemistry and Laboratory Medicine (IFCC)-aligned. The Hospital de Clínicas de Porto Alegre Clinical Pathology Department is a participant in and meets the standards of the HbA_1c_ external quality assurance program [24].

Based on HbA_1c_ levels, patients were categorized according to ADA criteria as having normal glucose tolerance (<5.7%), prediabetes (5.7–6.4%), or DM (≥6.5%) [11]. Patients with a known history of DM were classified as having DM regardless of HbA_1c_ levels. Patients were divided into four BMI categories, according to World Health Organization definitions: underweight (BMI <18.5 kg/m^2^), normal weight (BMI 18.5–24.9 kg/m^2^), overweight (BMI 25–29.9 kg/m^2^), class I and II obesity (BMI 30–39.9 kg/m^2^), and extreme obesity (BMI >40 kg/m^2^) [25].

### Data analysis and statistical methods

Statistical analyses were performed using PASW Statistics 18 and R 2.13.2 (R Foundation for Statistical Computing, Vienna, Austria). Data are presented as means ± standard deviation, median (interquartile range), or absolute and relative frequencies (%). To compare demographic, clinical and laboratory data, the Student *t-*test for independent samples, Mann–Whitney U, or chi-square tests were used as appropriate. Correlations were assessed with the Pearson or Spearman correlation coefficients. Kaplan–Meier event-free survival curves were used to compare the probability of in-hospital mortality according to different BMI strata. Cox regression models and the additional fit of cubic splines with four knots were used as exploratory data analysis for visual assessment of the functional relationship between BMI, HbA_1c_ and mortality while adjusting for APACHE II score [26-28]. The tested cutoff points for BMI were from 20 to 35, with increments of 0.5. For each increment, the study sample was divided into two groups defined by being lower or higher than the cutoff point and Cox proportional hazards models were used to estimate the risk of in-hospital mortality (dependent variable) for each stratum. The spline was then adjusted with the estimated risks to provide a better understanding of the relationship with BMI and HbA_1c_ levels. In addition, 95% confidence intervals for the fitted curve were calculated with a Bonferroni correction. A two-tailed *P* value <0.05 was considered significant.

## Results

### Patient profile

The study group comprised 199 patients, of whom 111 (55.8%) were men and 88 (44.2%) were women. The overall in-hospital mortality rate was 43.2% during a median of 16 (8–28) days of follow-up. Table 1 lists the characteristics of all study patients and compares survivors and nonsurvivors. The two groups did not differ regarding gender, comorbidities, history of DM, glucose tolerance, or HbA_1c_ levels. The number of days in hospital prior to ICU admission, APACHE and SOFA scores and plasma lactate levels, as well as the proportion of patients requiring mechanical ventilation, hemodialysis and vasopressor support, were higher among nonsurvivors than among survivors. Age and C-reactive protein levels also tended to be higher, while BMI was lower in nonsurvivors in comparison to survivors.

**Table 1:** Clinical and laboratory characteristics according to survival

	**Survivors**	**Nonsurvivors**	**P**
	**(n = 113)**	**(n = 86)**	
Age (years)	56 ± 17.4	61 ± 17	0.052
Males	65 (57. 5)	46 (53.5)	0.570
Days in hospital before ICU admission	2.5 (1–8)	6 (1–16)	<0.001
APACHE II	17.6 ± 7.9	24.8 ± 7.8	<0.001
SOFA	5 (2– 9)	8 (6 –13)	<0.001
Comorbidities			
COPD	20 (18)	12 (14)	0.443
CHF	12 (10.8)	9 (10.5)	0.938
HIV	6 (5.4)	7 (8.1)	0.443
Body mass index (kg/m^2^)	27.2 ± 7.3	24.7 ± 5	0.031
History of DM	26 (23)	24 (27.9)	0.430
Glucose tolerance			0.359
Normal	51 (45.1)	41 (47.7)	
Prediabetes	40 (35.4)	23 (26.7)	
Diabetes	22 (19.5)	22 (25.6)	
HbA_1c_ (%)	5.7 (5.3–6.3)	5.8 (5.2–6.5)	0.729
Serum glucose (mg/dL)	122 (99–160)	120 (95–167)	0.909
Lactate (mg/dL)	9.0 (6.3–15.3)	18 (0–35)	<0.001
C-reactive protein (mg/dL)	87 (27.5–150.4)	102 (44 –234)	0.067
Mechanical ventilation	69 (61.1)	68 (79.1)	0.008
Hemodialysis	15 (13.3)	30 (34.9)	<0.001
Vasopressor support	39 (34.5)	64 (74.4%)	<0.001

Data are expressed as mean ± standard deviation, median (interquartile range), or n (%). COPD, chronic obstructive pulmonary disease; CHF, congestive heart failure; HIV, human immunodeficiency virus. To convert glucose from mg/dl to mmol/L, multiply by the factor 0.005. To convert lactate from mg/dL to mmol/L, multiply by the factor 0.111.

A total of 149 (74.9%) patients had no known history of DM. However, a significant proportion of these seemed to have previously abnormal glucose metabolism (51 [34.2%] with prediabetes and 16 [10.7%] with DM). BMI was indicative of normal weight in 43.7% of patients. Excess weight was observed in 50.3% of the sample (33.1% overweight, 13.2% obese, and 4% extremely obese), whereas 6% were underweight.

### Relationship between glycemic control before ICU admission, total hemoglobin, body size, and prognostic factors for mortality

Because the relationship between HbA_1c_ and mortality could be confounded by BMI, anemia or other factors, the correlations of HbA_1c_ with total hemoglobin, BMI, and severity scores were analyzed. There was a weak correlation of HbA_1c_ with hemoglobin levels (r^2^ = 0.026; *P* = 0.025) and BMI (r^2^ = 0.05; *P* = 0.007). HbA_1c_ was not related to plasma lactate (r^2^ = 0.01; *P* = 0.135) or C-reactive protein levels (r^2^ < 0.01; *P* = 0.050), nor to APACHE II (r^2^ < 0.01; *P* = 0.958) and SOFA scores (r = 0.01; *P* = 0.449).

Body size, as estimated by BMI, was also not related to plasma lactate (r^2^ < 0.01; *P* = 0.352), C-reactive protein (r^2^ = 0.01; *P* = 0.196), APACHE II (r^2^ < 0.01; *P* = 0.334), or SOFA (r^2^ < 0.01; *P* = 0.634) scores.

### Relationship between HbA_1c_, body size and morbidity

Using HbA_1c_ to classify patients as having normal glucose tolerance, prediabetes, or DM, we were able to analyze whether increasingly abnormal glucose metabolism was related with increasing ICU morbidity. There were no glucose metabolism-related differences in the need for mechanical ventilation (normal vs. prediabetes vs. DM, *P* = 0.894), vasopressor support (normal vs. prediabetes vs. DM, *P* = 0.460), or renal replacement therapy (normal vs. prediabetes vs. DM, *P* = 0.583). These requirements also did not differ from the lowest to the highest BMI strata.

### Relationship between chronic glycemic control, body size, and mortality

There was no difference in HbA_1c_ levels between survivors and nonsurvivors (Table 1). When only the subgroup of patients with a known history of DM were analyzed, there was also no difference in HbA_1c_ levels between survivors and nonsurvivors (6.70% [5.8–8.5] vs. 6.8% [5.9–8.1]; *P* = 0.846).

**Figure 1:**
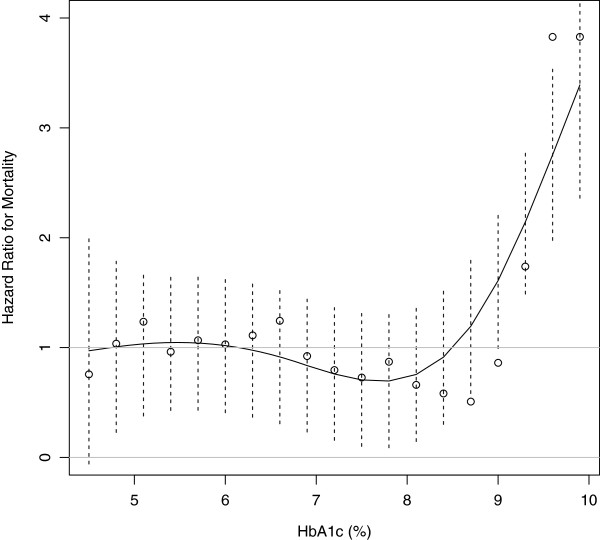
**Adjusted hazard ratio for hospital mortality at various HbA**_**1c **_**levels.** Hazard ratios >1 are indicative of higher risk for those with higher HbA_1c_ levels. Dashed lines represent the 95% confidence interval for each HbA_1c_ level. Results were obtained by multivariate Cox regression with cubic splines with four knots of HbA_1c_ adjusted for APACHE II scores.

**Figure 2:**
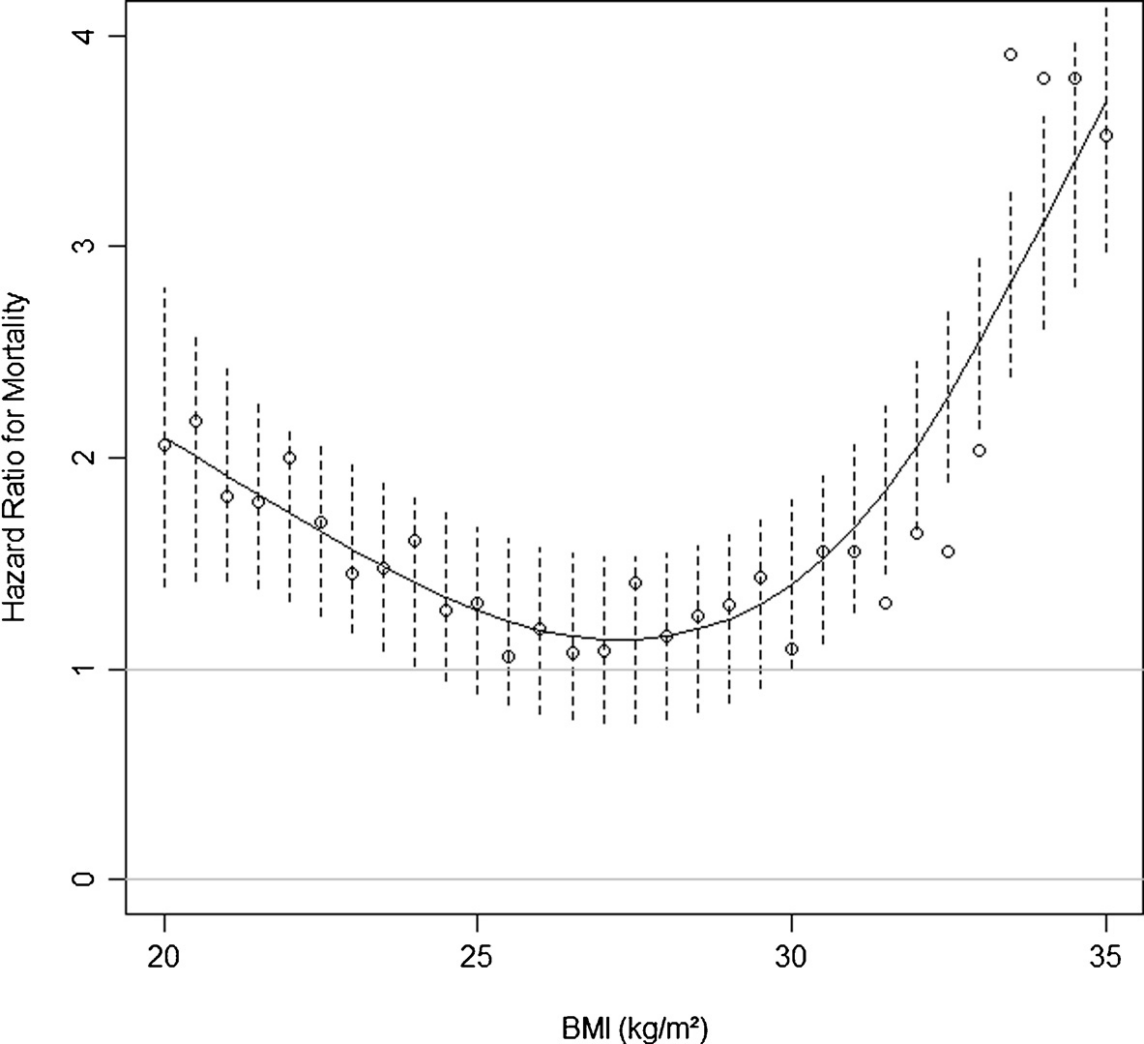
**Adjusted hazard ratio at various BMI levels.** The risk of hospital mortality is found when the hazard ratio expressed as mean (empty circle) and 95% confidence interval (dashed lines) do not cross 1. A hazard ratio >1 is indicative of increased risk for those with BMI lower than that chosen for analysis.

Cubic spline analysis was used to better analyze the relationship between HbA_1c_, BMI, and hospital mortality during follow-up. This statistical approach enabled graphical quantification of how the risk of hospital mortality (expressed as hazard ratio [HR]) varied across different HbA_1c_ and BMI levels. The risk of hospital mortality was significantly increased in patients with HbA_1c_ levels ≥9.3% compared with those with lower HbA_1c_ levels (Figure 1). Nevertheless, the relationship between BMI and risk of hospital mortality was nonlinear. Patients with a BMI between 24 and 30 kg/m^2^ did not exhibit increased hospital mortality (Figure 2). In-hospital mortality was higher among those with BMI in the 20–24 kg/m^2^ range and lower among those with BMI >30 kg/m^2^. For example, at lower body sizes, patients with BMI <23 kg/m^2^ had a 40% higher risk of in-hospital mortality (HR = 1.4) as compared with those with BMI ≥23 kg/m^2^. In patients with higher body size, those with BMI <33 kg/m^2^ had twice the risk of in-hospital mortality (HR = 2.0) compared with those with BMI ≥ 33 kg/m^2^. The lowest in-hospital mortality was observed among the patients with the highest BMIs (Figure 2).

**Figure 3:**
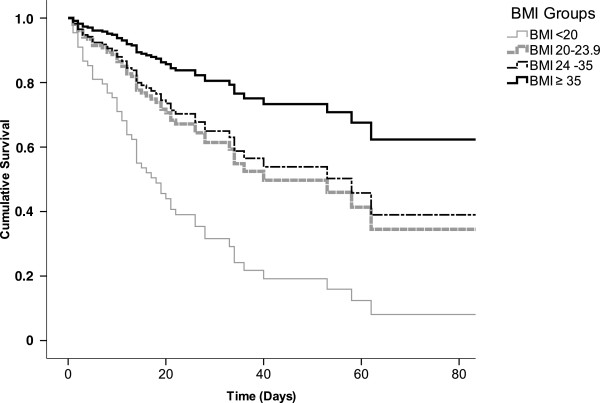
**Survival curves stratified by BMI.** Kaplan–Meier survival curves stratified by BMI and adjusted for APACHE II scores and HbA_1c_. BMI <20 vs. 20–23.9 kg/m^2^, *P* = 0.032; BMI <20 vs. 24–34.9 kg/m^2^, *P* = 0.010; BMI <20 vs. ≥35 kg/m^2^, *P* = 0.032.

Based on these findings (Figure 2), we stratified the study sample into four groups by BMI (group 1, BMI <20 kg/m^2^; group 2, 20–23.9 kg/m^2^; group 3, 24–34.9 kg/m^2^; group 4, ≥35 kg/m^2^) and compared them by multivariate Cox regression analysis. In comparison with group 1, mortality progressively decreased with increasing BMI while adjusting for HbA_1c_ and APACHE II score (*P* = 0.032; Figure 3).

## Discussion

Our findings suggest a nonlinear relationship between glycemic control before ICU admission, body size, and in-hospital mortality. The risk of the in-hospital mortality significantly increases with HbA_1c_ levels above 9.3%. It also progressively increases in patients with a BMI below 24 kg/m^2^, having no association with mortality in overweight patients, while progressively declining in those with a BMI above 35 kg/m^2^. The weak correlation between HbA_1c_ and BMI suggests an independent effect of these metabolic parameters as prognostic factors in critically ill patients.

Previous studies suggest that increased HbA_1c_ levels are a negative prognostic marker in patients with DM admitted to an ICU [12]. As DM is usually asymptomatic in its early stages, many patients may not be aware of this condition on ICU admission [29]. In our sample, 44.9% of participants with abnormal glucose metabolism had no previous history of prediabetes or DM. In a retrospective study, preexisting hyperglycemia affected the relationship between acute blood glucose levels and mortality, suggesting a significant interaction between chronic and acute glycemic control [9]. In our study using Cox regression models incorporating a cubic spline with four knots, we were able to detect an increased risk of mortality in those with significant hyperglycemia (HbA_1c_ above 9.3%). However, in the retrospective study, HbA_1c_ levels >7.0% with less stringent glycemic control were associated with decreased mortality [9].

Although our data do not suggest that body size interacts with past glycemic control to determine hospital mortality, BMI was significantly associated with mortality. Our findings confirm those of other studies that suggest an inverse relationship between body size and mortality in critically ill patients [15,16,30,31]. We analyzed whether this finding could be explained by other factors such as the requirement for mechanical ventilation, renal replacement therapy or vasopressor support, and our data suggest the BMI/mortality relationship is independent of these interventions.

Explanations for decreased mortality in obese patients admitted to the ICU are unclear. One hypothesis is that obese patients have a lower threshold for ICU admission as compared with normal-weight patients. Consequently, obese patients may have a better prognosis at ICU admission. However, we found that mortality differences were still present and obesity was still associated with protection against in-hospital death after adjusting for APACHE II scores. We are aware that APACHE may be artificially elevated in obesity (increased creatinine levels despite normal renal function, and decreased oxygenation because of lung atelectasis); however, there are no prognostic scores validated for use in the obese critically ill. Cubic spline analysis, which allowed analysis of how the risk of hospital death varied along the BMI distribution of the study sample, suggests that this relationship is not linear.

However, the high mortality for underweight and low-normal weight patients might be explained by the previous nutrition status, which is a finding more consistent in the literature [15,30], and suggests that special attention to the nutrition of this group of patients is needed.

There are some limitations to this study. First, HbA_1c_ levels were available to all healthcare providers involved in the care of the study patients and whose actions could affect patient management. However, at the study hospital, ICU staff follow a protocol with very well-known targets for acute glycemic control, as proposed in recent guidelines, which do not routinely take HbA_1c_ into account [32]. Second, body size was measured by the team involved in patient care at the ICU, which may have resulted in some variability in body size measurement. Although we did not perform a more sophisticated analysis of body size composition, such as bioelectric impedance, BMI was measured upon admission at ICU, therefore probably reducing the impact of fluid overload in body size determination. Moreover, the staff are well trained in measuring weight and height. Third, few patients had extreme obesity, which hindered quantification of the impact of BMI >40 kg/m^2^ on mortality. However, a recent study suggested no increased risk of mortality in patients with extreme obesity versus those with normal weight [33]. Fourth, we had a high mortality rate, as would be expected for severely ill patients. The expected mortality for our median APACHE II score of 21 is 38%, which was similar to the study overall hospital mortality of 43.2% [22].

## Conclusions

This study has shown that preexisting hyperglycemia (HbA_1c_ >9.3%) increases the risk of hospital mortality in critically ill patients. Moreover, underweight and low-normal weight patients exhibited a decreased survival rate compared with those who were overweight. Obesity, however, was associated with decreased mortality in critical ill patients. As the prevalence of DM and obesity continue to increase globally, intensivists will require a better understanding of the impact of these conditions in the ICU setting, and will need to develop strategies to improve the care of these patients. Further studies are required for the construction of a strategy for the management of patients with hyperglycemia and obesity in the ICU.

## Abbreviations

APACHE II: Acute physiology chronic health evaluation II; BMI: Body mass index; DM: Diabetes mellitus; HbA_1c_: Glycated hemoglobin; ICU: Intensive care unit; SOFA: Sequential organ failure assessment.

## Competing interests

All authors declare no conflict of interest.

## Authors’ contributions

MVV had full access to all of the data in the study and takes responsibility for the integrity of the data and the accuracy of the data analysis. Study concept and design: MVV, SRV, JLG, and FG. Data collection: MVV, RBM, ARF, MSF and FG. Data analysis and interpretation: all authors. Manuscript Drafting: all authors; Critical revision of the manuscript for important intellectual content: all authors; Statistical analysis: MVV, VBLT, FG. Obtained funding: MVV, JLG and FG. All authors read and approved the final manuscript.

## Pre-publication history

The pre-publication history for this paper can be accessed here:

http://www.biomedcentral.com/1472-6823/14/50/prepub
